# Saccade-Related Activity in Superior Colliculus Predicts Eye Choice for Goal-Directed Saccades in Monkeys With Strabismus

**DOI:** 10.1167/iovs.66.14.46

**Published:** 2025-11-18

**Authors:** Jérome Fleuriet, Tiphaine Belloir, Mark M. G. Walton

**Affiliations:** 1 Washington National Primate Research Center, University of Washington, Seattle, Washington, United States; 2 Department of Intensive Care, AP-HP University Versailles Saint Quentin-University Paris Saclay, Garches, France

**Keywords:** strabismus, superior colliculus, saccade, exotropia, esotropia

## Abstract

**Purpose:**

Infantile strabismus syndrome is a common disorder characterized by a chronic misalignment of the eyes that is present in infancy. The disorder is associated with a wide range of abnormalities including severe impairments of binocular vision, impaired depth perception, impaired motion perception, amblyopia, nystagmus, a loss of disparity vergence, asymmetrical smooth pursuit gain, and saccade disconjugacy. The chronic inability to direct both eyes to the same visual target forces the brain to decide which eye to bring to any given object of interest in the visual field. We wondered if different populations of saccade-related neurons in superior colliculus might be activated, depending on which eye is to be brought to the target. We hypothesized, therefore, that the height of the movement field peak might differ for right-eye-to-target versus left-eye-to-target saccades.

**Methods:**

This study used single-unit, extracellular recording to investigate the bursts of saccade-related neurons in the superior colliculus in a nonhuman primate model of this disorder. Movement fields were plotted separately for saccades that brought the left eye or right eye to the target. Several statistical methods were used to compare the height of the peak between conditions.

**Results:**

A majority of the isolated neurons showed significantly stronger bursts when a particular eye was directed to a visual target, compared to when the fellow eye was brought to the target.

**Conclusions:**

These results imply that, in monkeys with strabismus, different (but overlapping) populations of burst neurons are active depending on which eye is directed to a visual target.

Infantile strabismus syndrome is a very common disorder, characterized by a long list of deficits in visual and oculomotor functions.^1^^–^^4^ Deficits involving eye movements include a severe impairment of vergence (including a complete loss of disparity vergence),^5^^,^^6^ and asymmetrical smooth pursuit gain.^7^ Saccades in this disorder are typically disconjugate in terms of direction and amplitude;^8^^–^^12^ even though the goal-directed saccade is accurate. More recently, evidence has emerged that saccade durations and the curvature of trajectories don't always match for the two eyes.^13^

An important consequence of these abnormalities is a severe, chronic impairment in the ability to simultaneously direct both eyes to the same visual target. Thus, when shifting gaze, the brain must decide *which* eye to bring to the new target. An additional complication is the fact that, because of the disconjugacy of saccades, different commands are needed to satisfy a given desired displacement, depending on which eye is to be brought to the target. This is a computational problem that does not typically exist for normal primates, who almost always bring both eyes to the same target, even in the case of disjunctive saccades between targets that differ in both direction and distance. Figure 1 shows this problem schematically. In all three panels, the target steps 10° to the right, so the desired displacement is identical when each eye is fixating the center of the screen. Because the saccades are disconjugate, however, the same command that is appropriate when the right eye is brought to the target (Fig. 1A) will be inappropriate when the left eye is brought to the target (Fig. 1B). In reality, subjects with strabismus are able to make accurate saccades with either eye,^12^ as shown in Figures 1A and 1C. If one compares Figures 1A and 1C, however, it is clear that the brain must generate different saccadic commands to satisfy the 10° rightward desired displacement, depending on which eye the animal intends to bring to the target. This implies that, in strabismus, the brain somehow carries information about which eye should be brought to a visual target of interest.

**Figure 1.**
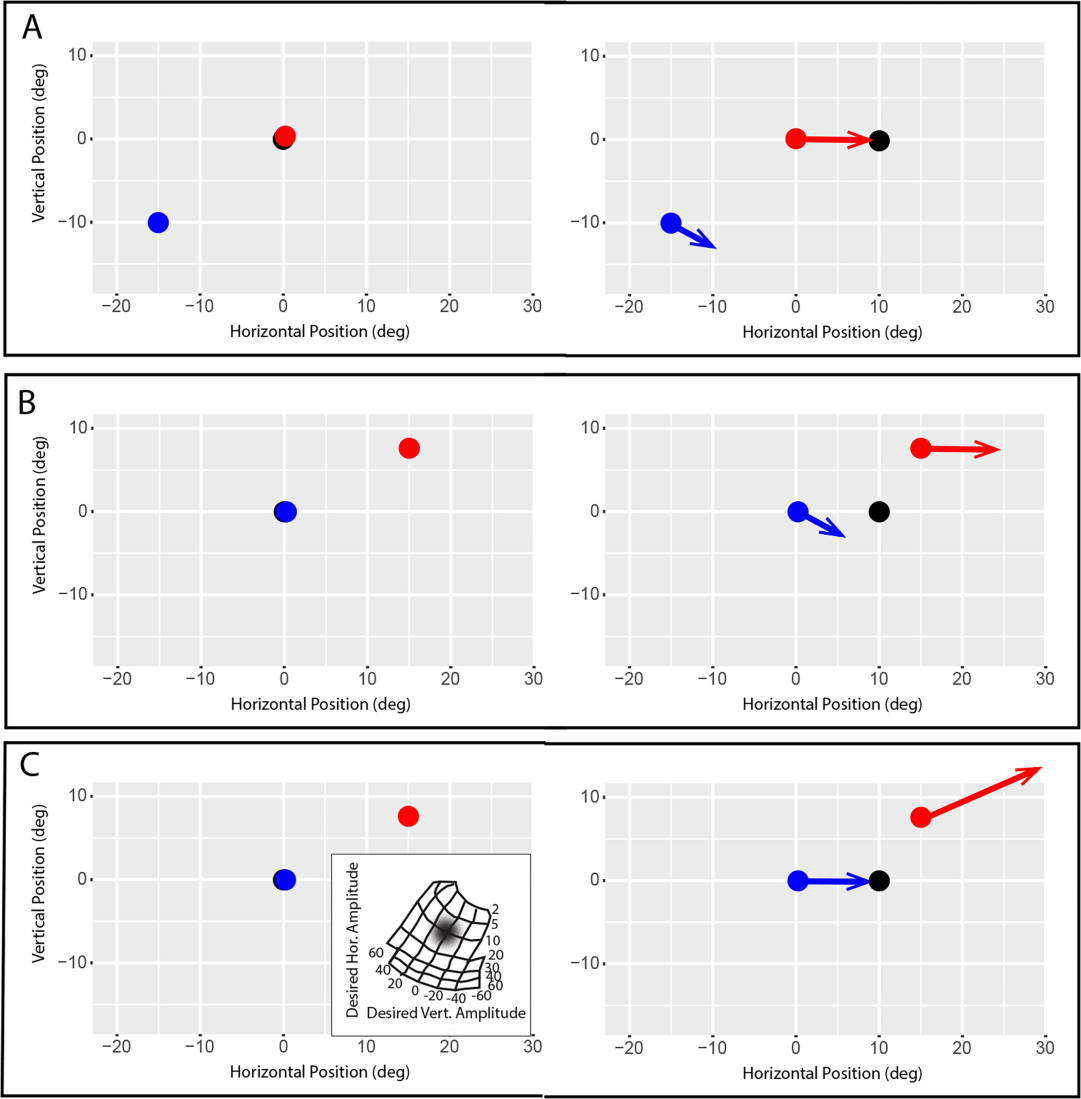
Schematic representation of the orienting problem that the brain must solve, in strabismus, to ensure that either eye can be brought to a visual target despite saccade disconjugacy. *Black filled circle:* location of the visual target in the target step task. *Red filled circle:* location of the right eye. *Blue filled circle:* location of the left eye. **(A)** Hypothetical trial in which the right eye is directed to the target. In the right panel, the target steps 10° to the right. The subject then makes a saccade that successfully brings the right eye to the new location of the target. **(B)** Another hypothetical trial in which the task is the same but the subject is using the left eye to fixate the target before it steps. Because of saccade disconjugacy, if the brain generates the same saccadic command after the target steps 10° to the right, the left eye will miss the target. Note that this is not what happens. **(C)** Instead, subjects with strabismus are typically able to successfully bring either eye to the target. Note that the brain must generate a different command to satisfy the same desired displacement, depending on which eye is brought to the target.

The development of non-human primate models for infantile strabismus syndrome has permitted the investigation of neurophysiological abnormalities in this disorder.^1^^,^^2^ Classic experiments have demonstrated that prolonged deprivation of binocular vision in early postnatal life in monkeys leads to a permanent strabismus with characteristics that are an excellent match for the clinical syndrome observed in human children with this disorder.^5^^,^^14^ Decades of work in monkeys with experimentally induced infantile strabismus has revealed a widespread loss of binocular responses in visual cortical areas.^15^^–^^19^ This, in turn, disrupts the development of normal tuning in brainstem areas involved in oculomotor control.^1^^,^^2^ For example, in monkeys with strabismus, near and far response neurons in the supraoculomotor area that would normally encode disparity vergence instead modulate their discharge, depending on which eye is used to view the target.^20^^,^^21^ This body of literature suggests the possibility that abnormally monocular tuning in visual cortical areas alters the developmental trajectories of neurons in oculomotor brain regions.

In the oculomotor neurophysiology literature over the past few decades, a number of studies have reported that, in normal primates, saccade-related neurons can carry signals that are more predictive of the movement of a single eye.^22^^–^^27^ These studies suggest the possibility that, in primates with strabismus that are unable to direct both eyes to the same target, monocular saccade-related neurons might burst more strongly if their “preferred” eye is the one that is to be brought to the target.

The superior colliculus (SC) is a visuomotor layered structure in the midbrain that plays a crucial role in the selection of targets for saccadic eye movements and in the preparation of a sequence of two or more saccades.^28^^–^^35^ Neurons in the intermediate and deep layers are topographically organized and characterized by saccade-related bursts of spikes associated with gaze shifts of a particular vector (direction and amplitude).^36^^–^^38^ When a delay is imposed between the appearance of a visual target and the signal to initiate the saccade, some SC neurons exhibit both visual and saccade-related bursts and some show tonic prelude activity during the delay period.^30^^,^^31^^,^^38^^,^^39^ Neurophysiological studies, conducted in normal animals over the past few decades, have led to the view that at least some of these neurons carry signals related to desired displacement.^40^^,^^41^ Because monkeys with strabismus can only bring one eye to a visual target, we wondered whether SC neurons in these animals might show a preference for saccades that bring a particular eye to the target. The present study was designed to test the hypothesis that, in monkeys with strabismus, a subset of neurons in SC discharge more strongly (i.e., more spikes in the burst, particularly for saccades near the movement field peak) for saccades that bring a particular eye to a visual target, compared to saccades that bring the other eye to the target.

## Methods

### Subjects and Surgical Procedures

Prior to the beginning of animal training, all training, surgical and experimental procedures were approved in advance by the Institutional Animal Care and Use Committee at the University of Washington and were in compliance with the National Institutes of Health Guidelines for the Care and Uses of Laboratory Animals and with the ARVO Statement for the Use of Animals in Ophthalmic and Vision Research.

Three monkeys were used as subjects in this experiment. Two (monkeys XT1 and ET1) were *Macaca mulatta* and one (Monkey XT2) was *Macaca nemestrina*. Monkeys XT1 and XT2 had large-angle, A-pattern exotropia resulting from a bilateral medial rectus tenotomy during the first week of life. Monkey XT1 (∼25° when viewing with the right eye and ∼35°–40° when viewing with the left eye) was the same individual that carried this designation in several of our previous studies;^8^^,^^42^^–^^44^ Monkey XT2 (typically ∼15°–35°) is the same individual as the similarly-designated monkey in our previous studies of interstitial nucleus of Cajal and nucleus prepositus hypoglossi.^43^^,^^45^ Monkey ET1 (typically ∼15° esotropia) wore prism goggles (20 PD base-in and 20 PD base-down) at all times for the first three months of life, which resulted in a permanent A-pattern esotropia.^8^^,^^42^^,^^44^ More detailed descriptions of the strabismus in these animals can be found in these studies.

All monkeys used in this study underwent two surgeries to prepare them for neurophysiological experiments. First, a titanium post was surgically affixed to the skull so that head movements could be prevented during experimental sessions. Eye coils were implanted underneath the conjunctiva of both eyes so that eye position could be measured with high spatial and temporal resolution using the magnetic search coil technique (CNC Engineering, Seattle, WA, USA).^46^^,^^47^ This method has been the gold standard for measurement of eye position in oculomotor research for more than 40 years because it yields measurements at high spatial and temporal resolution without affecting eye movements.^46^^,^^47^ A titanium recording chamber was affixed to the skull over a 16 mm craniotomy, at a location that permitted access to the SC. To ensure that the duration of each surgery was short enough to ensure the safety of the animal, two separate surgeries were used to install these three types of implant.

### Behavioral Tasks and Visual Display

Monkeys were trained to voluntarily enter a primate chair and to accept having their head movements restrained during data collection. The chair was clamped to a movable platform that docked into the base of a 1.5-meter coil frame, which ensured that the animal's eyes were always 57 cm from a tangent screen, onto which visual targets were back-projected. The animals were given applesauce rewards to encourage them to maintain fixation on a red visual target that consisted of a 0.25° laser spot. A system of galvanometers was used to control the location of the target.

Target position feedback signals and eye position signals from the two eye coils were passed through an anti-aliasing, six-pole Bessel filter. These signals were digitized at 1 kHz using CED-Power1401 hardware (Cambridge Electronic Design, Cambridge, UK). Monkeys with esotropia wore a set of liquid crystal shutter goggles (Micron Technology, Inc., Boise, ID), which allowed the experimenter to easily control which eye (or both) viewed the screen by flipping switches in the instrument rack. It is well known, however, that subjects with large angle infantile exotropia have a visual weakness for targets in the contralateral hemifield (i.e., images that fall onto the temporal hemiretina).^48^^–^^50^ This being the case, monkeys XT1 and XT2 (both of whom had large angle exotropia) were typically unable to perform the task if one eye was occluded and the target location was more than a few degrees into the contralateral visual hemifield. Both of these animals reliably used to the left eye to view targets >10° to the left of straight ahead and the right eye to view targets >10° to the right of straight ahead. Between −10° and 10°, these animals switched viewing eye frequently. Thus it was still possible to obtain an adequate data set, with each eye fixating, from the monkeys with large-angle exotropia.

#### Target Step Task

The target position was chosen by the computer, by randomly selecting horizontal and vertical coordinates from a user-selected list (−22° to 22° in 2° or 3° increments, both horizontally and vertically). After a time interval that varied between 1.5 and 5 seconds, the target stepped to a new, randomly selected location. Because the target could step directly between any two locations, rather than returning to a central location (i.e., 0,0), this task could potentially elicit large saccades of 40° or more in almost any direction (although very large saccades like that were uncommon).

### Unit Recording and Localization of SC

Tungsten and glass microelectrodes (Frederick Haer, Bruswick, ME, USA) were used to record single-unit, extracellular activity for this study. The SC was identified by single- and multi-unit activity that is characteristic of this structure, such as bursts of spikes associated with the appearance of a visual target or saccades of a particular vector. Typically, visual neurons were encountered first, while driving through the superficial layers. In most cases the saccade-related activity was observed after driving another 1.2 to 2 mm after the first visual activity was heard. When an isolated neuron appeared to burst in association with saccades, all targets were switched off long enough to verify that bursting could be heard associated with saccades in complete darkness. In some cases, microstimulation (0.1 ms per pulse, 7–40 µA, 400 Hz, 200–300 ms train duration) trains were used, to evoke the “staircase” saccades (at least two saccades per train) that are characteristic of stimulation of the saccade-related layers of SC. Some of the microstimulation data were used for a previously published study.^51^

### Data Analysis

During recording sessions, the experiment was controlled using Spike 2 software (Cambridge Electronic Design, Cambridge, UK). This same software was also used for preliminary offline analyses, such as visualization of eye position traces and unit activity. After the unit isolation was confirmed in Spike 2, data were exported into Matlab (Mathworks, Natick, MA, USA), where more extensive analyses were conducted using custom functions written by the authors. Next, offline spike detection was performed using a custom algorithm that we have used in previous studies.^44^^,^^52^^,^^53^

Seven-point parabolic differentiation of instantaneous eye position was used to estimate the instantaneous vertical and horizontal eye velocities. Saccade onsets and offsets were identified using a custom algorithm that was based on a combination of vectorial velocity and acceleration criteria.^8^

For each detected saccade, the number of spikes was counted within a time window that began 20 ms before saccade onset and ended at saccade offset. This time-window approach was deemed necessary, rather than using a spike frequency threshold-based algorithm, because we were testing the hypothesis that some SC neurons burst weakly, or not at all, for movements that bring a particular eye to the target. Fixation switching saccades were defined as those for which a different eye was on target before and after the movement. Fixation-switching saccades were defined as those for which a different eye was on target before the target step and after the saccade to re-acquire the target after the step. These movements were excluded from subsequent analyses.

In 2003 Walton and Mays^54^ reported that, in normal monkeys, the movement field peaks of SC neurons are often in different locations depending on whether the saccade is accompanied by a change in vergence angle. Oddly enough, the direction and size of the shift in peak location was highly idiosyncratic. Given this result, we felt that it was important to assess the height of movement field peaks in a way that would be unaffected by any possible changes in the *location* of the peak. One approach we took was to simply compare the number of spikes in the burst for the 10% of trials with the largest number of spikes, which is very similar to the approach we took in our 2003 SC article.^54^ There are limitations associated with this method, however, such as the assumption that the number of trials near the peak is similar between conditions. Although this is a reasonable assumption most of the time, we judged it important to use other methods as well.

It has been known since the 1970s that the movement fields of SC neurons are approximately “hill-shaped”, which inspired many authors to use two-dimensional Gaussian functions to describe them and model them.^55^^–^^57^ With this in mind, movement field plots were constructed, using horizontal and vertical saccade amplitude to predict the number of spikes in the burst. Using Matlab's curve-fit tool, these data were plotted separately for left-eye-to-target and right-eye-to-target saccades and the data fit with a two-dimensional Gaussian function (Equation 1):

(1) $$\begin{array}{c} f\left(x,y\right)=\;\frac{1}{2\pi \sigma^{2}}e^{-\left[{\left(x-\mu_{x}\right)}^{2}+{\left(y-\mu_{y}\right)}^{2}\right]/\left(2\sigma^{2}\right)} \end{array}$$

where *x* is the horizontal amplitude of the saccade (in degrees), *y* is the vertical amplitude of the saccade (in degrees). This procedure yields parameters related to the horizontal and vertical locations of the movement field peak, and the height of the peak. The curve fit tool also yields 95% confidence intervals for each of these parameters. These movement fields were also plotted separately for the right and left eyes for each viewing eye condition. The height of the movement field peak was compared for left-eye-to-target versus right-eye-to-target saccades; if the confidence intervals did not overlap then the neuron was considered to have statistically significant viewing eye selectivity. A monocularity index (MI) was computed for each neuron, using Equation 2:


(2) $$\begin{array}{c} MI=\;\frac{\left(Peak_{IpsilateralEye\;}-\;Peak_{ContralateralEye\;}\right)}{\left(Peak_{IpsilateralEye\;}+\;Peak_{ContralateralEye\;}\right)} \end{array}$$

A neuron that bursts only for the ipsilateral or contralateral eye would have a MI of 1 or −1, respectively. A neuron with no viewing eye selectivity at all would have a MI of 0.

Finally, we directly compared the number of spikes for left-eye-to-target saccades to right-eye-to-target saccades, with the vector of the viewing eye similar in both conditions. While this method could, potentially, be misleading if the location of the peak shifts between conditions the potential limitations of these three methods are not the same. Thus, if all three give a similar answer, we would take this as convincing evidence that the effect is real.

## Results

**Figure 2.**
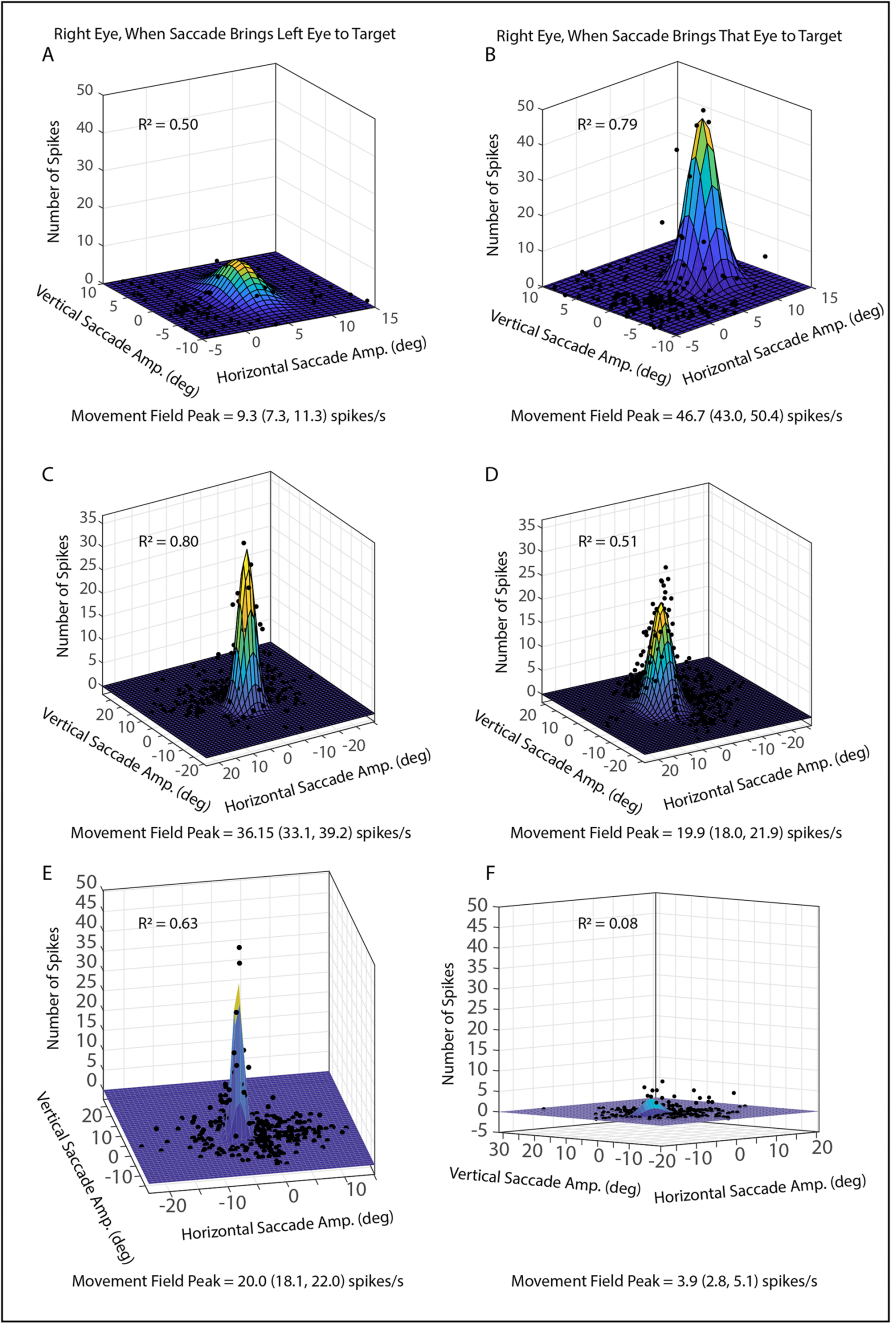
Comparisons of movement fields of SC neurons when the monkey brings the left eye (*left column*) or right eye (*right column*) to the target. Data are fit with two-dimensional Gaussian functions. The neuron shown in the top row exhibits only a very weak response when the left eye is brought to the target, even for saccades near the movement field peak **(A)**. The same neuron, however, exhibits a vigorous bust of spikes for saccades near the movement field when the right eye is brought to the target **(B)**. The neuron shown in the middle row, on the other hand, shows a robust burst of spikes for preferred-vector saccades, regardless of which eye is brought to the target. However, the Gaussian peak is significantly higher for left-eye-to-target saccades **(C)** than for right-eye-to-target saccades **(D)**. The neuron shown in the bottom row shows a robust burst for left-eye-to-target saccades **(E)** but barely responds at all for right-eye-to-target saccades **(F)**. However, for all three example neurons, that the *R*^2^ values are much higher for the preferred eye compared to the non-preferred eye.

The basic data set consisted of 48 well-isolated neurons, recorded from the SC. Of these, six were excluded from saccade-related analyses because they turned out to be visual neurons. Another six were excluded from saccade-related analyses because isolation was lost before we were able to collect a sufficient number of trials to plot movement fields with each eye viewing. Finally, four neurons turned out to have open movement fields (or the peak was outside the range of data collection). This left 32 saccade-related neurons that were analyzed using the Gaussian fits. Figure 2 shows example movement field plots for the right eye for three example neurons, including one recorded from left SC in monkey XT1 (Figs. 2A, 2B) and two recorded from right SC in monkey ET1 (Figs. 2C–F). For the neuron shown in Figures 2A and 2B, when the saccade brings the right eye to the target (Fig. 2B), the neuron shows a robust burst (>40 spikes) for rightward saccades of approximately 7°. For saccades that bring the left eye to the target, however, the strongest bursts typically have fewer than 10 spikes (Fig. 2A). Similarly, the neuron shown in Figures 2E and 2F shows more spikes in the bursts for saccades that bring the left eye to the target. A more typical example is shown in Figures 2C and 2D: the peak of the Gaussian function is higher for saccades that bring the left eye to the target, but the neuron shows robust bursts regardless of which eye is brought to the target. Of the 32 neurons that were fit with Gaussian functions, the height of the movement field peak differed significantly between viewing eye conditions for 23 neurons (72%).

The above analysis seems to suggest that the height of the movement field peak may differ for some SC neurons, depending on which eye is brought to the target. However, there is another potential interpretation that must be considered. If the movement field is more disorganized (i.e., a given saccade vector is associated with a more variable number of spikes) when a particular eye is brought to the target, then the Gaussian fit will be poorer, which would tend to lead to a lower estimate of the peak height, even if the strongest bursts in the two conditions are similar. Indeed, one can see an example of this in Figures 2C and 2D. Although the Gaussian peak is clearly lower for right-eye-to-target saccades, there are a number of individual data points above the Gaussian peak. This raises the question of whether the bursts are truly stronger for left-eye-to-target saccades for this neuron.

If differences in the goodness of fit are a factor, one would expect to find a significant correlation between the monocularity index and the difference in *R*^2^ values (i.e., *R*^2^ for left-eye-to-target − *R*^2^ for right-eye-to-target). Indeed, this relationship was significant for both the right eye and the left eye (*R*^2^ = 0.50 and 0.30, respectively). Thus the neurons with the monocularity indexes that differed the most from 0 also tended to be the ones that showed the greatest difference in the variance accounted for for the two eyes.

**Figure 3.**
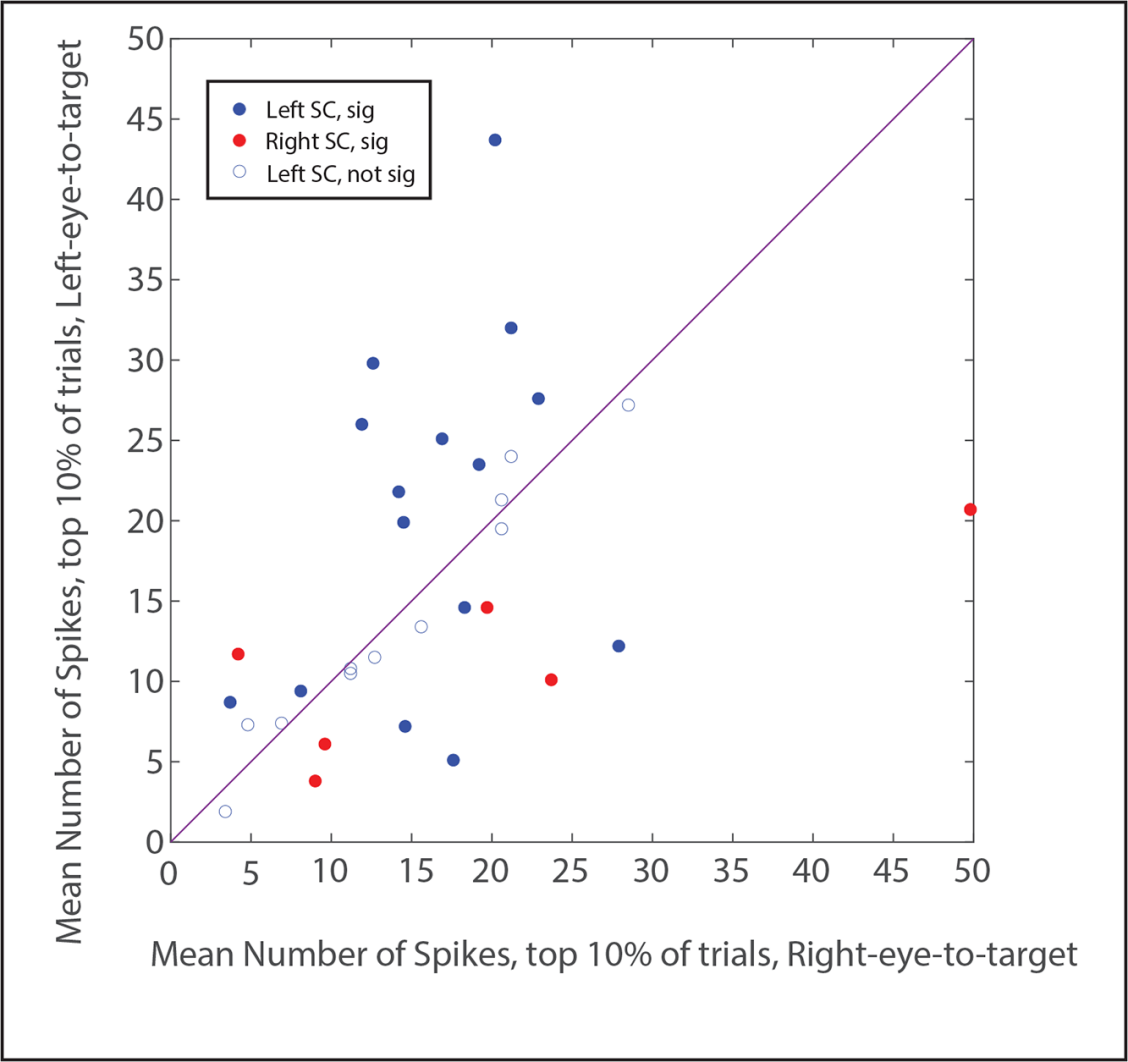
Comparison of the mean number of spikes for the 10% of trials with the largest number of spikes for each viewing eye condition (left-eye-to-target versus right-eye-to-target). Each data point represents a single neuron. *Blue* = left SC; *red* = right SC. *Filled circles* indicate that there was a significant difference between the two conditions; *open circles* indicate that there was no significant difference. The fact that most neurons showed a significant difference for the top 10% of bursts indicates that this effect is not *solely* a consequence of a better goodness of fit for one eye than for the other.

The above analysis suggests thus the possibility that the difference in movement field peak heights, as measured by the Gaussian curve fits, might be an artifact of a poorer fit for one eye. To investigate this question, the data were also analyzed using a method that is immune to the goodness-of-fit problem. In this analysis, the mean number of spikes was computed for the 10% of saccades with the largest number of spikes^54^ for right-eye-to-target versus left-eye-to-target saccades. Two-tailed *t*-tests were then used to compare the mean number of spikes for the top 10% of trials for these two conditions. The results were quite similar to those obtained from the curve fit approach, with a significant difference being found for 21/32 (66%) neurons. Figure 3 is a scatterplot that compares the mean number of spikes for left-eye-to-target and right-eye-to-target saccades, for the 10% of trials with the largest number of spikes. For both left and right SC, there was a tendency for neurons to show a significant preference for saccades that brought the ipsilateral eye to the target but that was not always the case (ipsilateral eye preference = 16 neurons; contralateral eye preference = 5 neurons). Thus it appears that, although the estimated heights of the movement peaks were likely affected by differences in the goodness-of-fit between conditions, this does not fully explain the observation that many of the saccade-related neurons in our sample burst more strongly for saccades that brought a particular eye to the target compared to saccades that brought the other eye to the target.

### Analysis of Matched-Vectors

**Figure 4.**
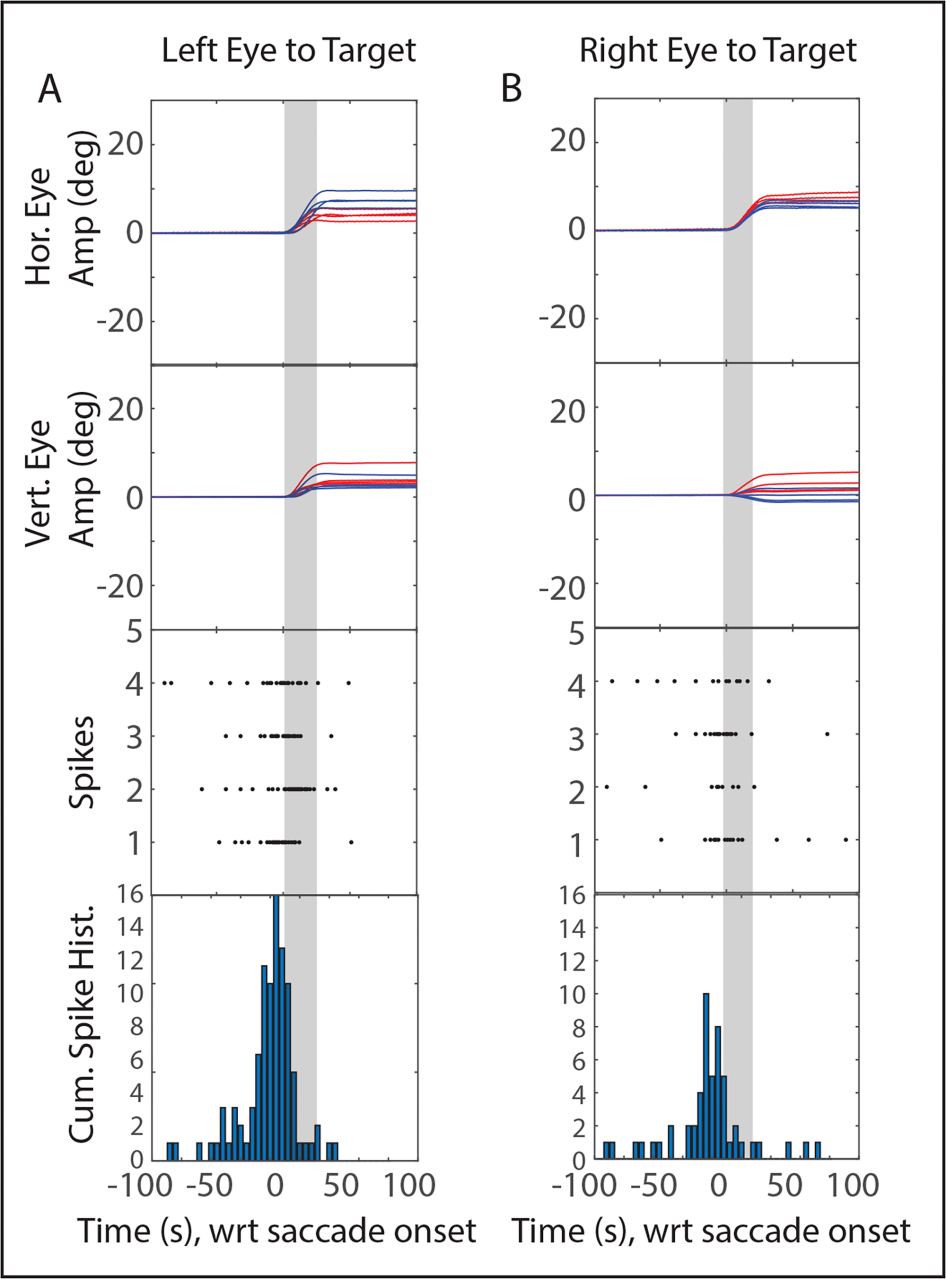
Eye position traces, rasters, and cumulative spike histograms for groups of saccades with similar vectors for left-eye-to-target **(A)** and right-eye-to-target saccades **(B)**. Note that the left eye's saccade vectors in **A** are quite similar to the right eye's saccade vectors in **B**, yet the saccade-related bursts are clearly stronger for the former than the latter. Also, please note that the initial eye position was not always the same: The y-axis of the traces in the top two rows thus represents component amplitude, not position.

Another way of comparing the activity level of saccade-related SC neurons, for right-eye-to-target versus left-eye-to-target saccades, would be to select matched vectors from the two conditions, focusing on trials very near the movement field peak.^58^ Because the data were not collected with this analysis in mind, there were not enough trials near the movement field peak for many of our recordings. We performed this comparison only if there were at least six trials in each condition with vectors within 3° of the peak (if the neuron preferred saccades of <8°) or 5° of the peak (if the neuron preferred saccades of >10° amplitude). If this criterion was met, two-tailed *t*-tests were then performed to compare the number of spikes in the burst for the two viewing eye conditions. Figure 4 shows eight example trials from a neuron that showed very little activity until shortly before saccades, including four that brought the left eye to the target (Fig. 4A) and four that brought the right eye to the target (Fig. 4B). Note that, because the saccades are disconjugate, the vectors of the non-viewing eye's saccades differ between the two panels, but the vectors are similar for the viewing eye (mean amplitudes for viewing eye: left eye: [7.9, 3.7]; right eye: [8.0, 5.1]). Nonetheless, it is clear that the neuron bursts more strongly if the saccade brings the left eye to the target. A significant difference was found for 11/15 neurons. Furthermore, the answer was the same as for the Gaussian fits for 10/15 of these neurons (either significant in the same direction for both or not significant for either analysis).

For the above analysis, one might object that matching the vectors of the viewing eye, for right-eye-to-target versus left-eye-to-target saccades means that the vectors for both the left and right non-viewing eyes will differ between conditions. With this objection in mind, we also performed a comparison of the number of spikes for pairs of saccades for which the vectors for *both* eyes were matched for the left-eye-to-target and right-eye-to-target conditions. Specifically, the vectors for both eyes had to match within a vectorial distance of 2°. If a given saccade from one condition matched multiple saccades from the other condition, only the closest match was used in this analysis. Additionally, to ensure that the comparison would only involve trials that fell within the response field of the cell, saccade pairs were only included in this analysis if there was a minimum of five spikes for at least one of the two conditions (left-eye-to-target or right-eye-to-target). Finally, this analysis was only performed on a given recording if a minimum of six pairs of vector-matched saccades were found that satisfied the above criteria (*n* = 29 neurons). The percentage of total trials that satisfied these criteria varied widely but was typically in the range of 8% to 20%.

For the matched pairs, a statistically significant difference was found between the mean horizontal amplitude for 24% of neurons and for the vertical amplitude for 28% of neurons. The actual vectorial differences were much smaller than the 2° tolerance window. The largest mean difference between the matched pairs was 0.74 but, across all the neurons for which this analysis was performed, the mean difference was quite small (0.28°). To investigate the relationship between these small vector differences and the differences in the number of spikes we also re-ran this analysis using a tolerance window of 1°. Not surprisingly, this reduced the number of neurons for which a sufficient number of matched pairs were obtained and reduced the statistical power of the tests so that fewer neurons showed a statistically significant difference in the number of spikes. However, even with this stricter inclusion criterion, 9/23 (39%) of neurons still showed a significant difference in the number of spikes between the right-eye-to-target and left-eye-to-target conditions. Of those nine, seven showed no significant differences between either the horizontal or vertical amplitudes for the matched pairs. For the 1° tolerance window the mean vectorial difference between the matched pairs was 0.17°.

It should be noted that the example neurons shown in Figures 2A, 2B, 2E, and 2F showed a significant difference in the vertical amplitudes for the matched pairs when the tolerance window was set to 2° (in fact, the neuron shown in Figs. 2E and 2F showed the largest mean difference in vertical amplitude for any neuron in our sample, 0.74°). When the tolerance window was set to 1°, however, neither one of these neurons showed any significant differences for either horizontal or vertical amplitudes, and the effect sizes were largely unchanged.

**Figure 5.**
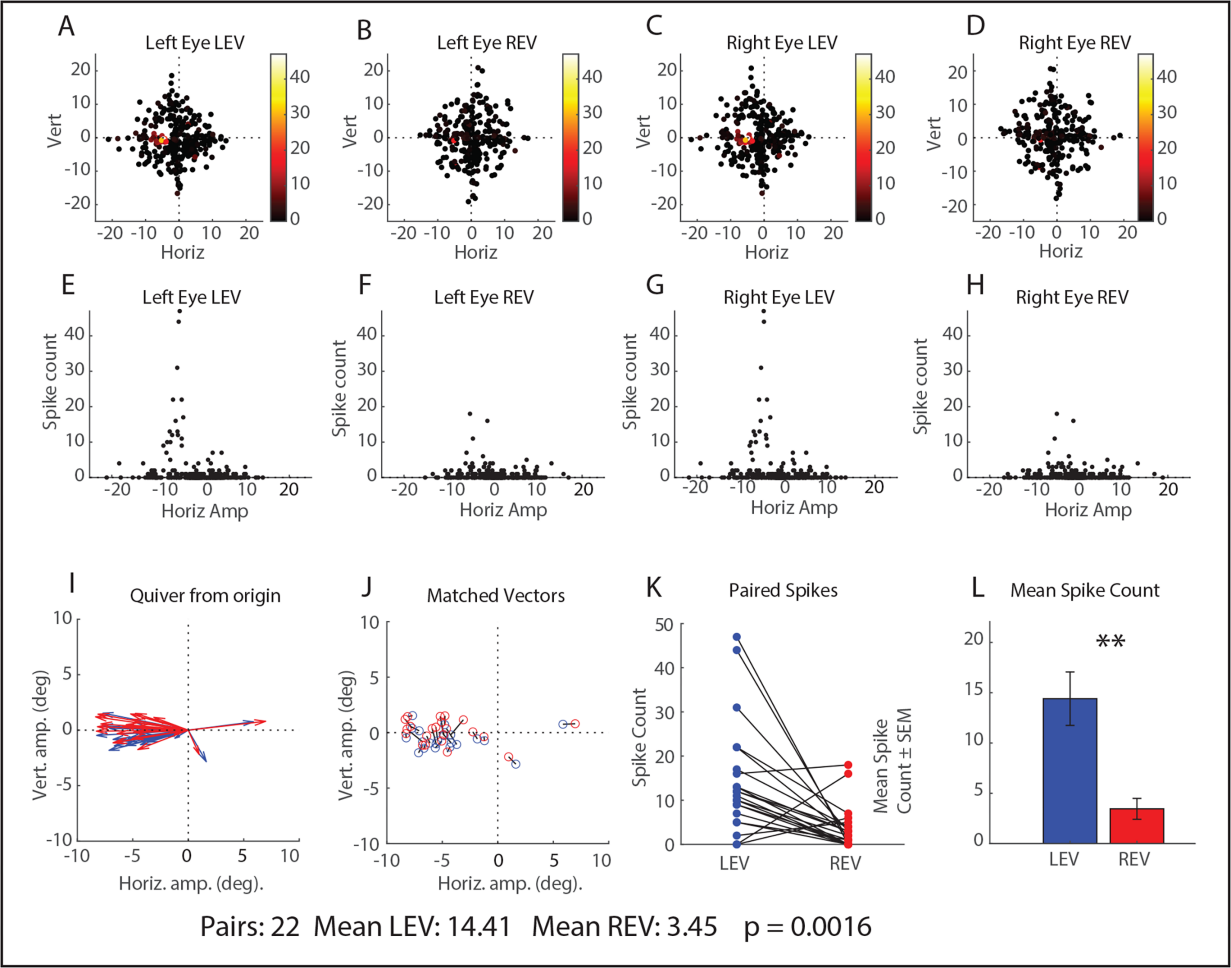
Data for the same example neuron shown in panels **E** and **F** of Figure 2. *Top row* **(A–D)**: horizontal and vertical amplitudes of all trials for this neuron. Each dot represents a single saccade, color-coded according to the number of spikes in the burst. Note that there are numerous trials at and near the movement field peak in both conditions. *Middle row* **(E–H)**: Scatterplots showing the number of spikes as a function of horizontal amplitude for each eye and for both conditions. Note that, even with no analyses, it is straightforwardly obvious that the strongest bursts contain more spikes in the left-eye-to-target condition, compared to the strongest bursts in the right-eye-to-target condition. *Bottom row* **(I–J)**: The vectors of the matched pairs that were included in this analysis. **(K)** The number of spikes for each condition for the matched-vector pairs. The *black lines* connect the values for matched vector pairs. **(L)** Bar graphs compare the mean number of spikes for matched-vector pairs for the two conditions.

Figure 5 shows results for the same example neuron shown in Figures 2E and 2F. The top row shows the horizontal and vertical amplitudes for each saccade, with the dots color-coded according to the number of spikes in the burst. These panels demonstrate that the range of data collection included the peak, with data points at the peak and all around the peak in both conditions. The middle row shows panels that plot the number of spikes as a function of horizontal amplitude for both conditions as this neuron show no preference for vertical amplitudes. From these panels, even in the absence of any statistical analyses, one can clearly see that the movement field peak is much higher for left-eye-to-target saccades than for right-eye-to-target saccades (Fig. 5E vs. 5H). The bottom row compares the number of spikes in the burst for pairs of saccades, vector-matched for both eyes. From this figure it is clear that, for the overwhelming majority of vector-matched saccades, there were more spikes for the left-eye-to-target condition than for the right-eye-to-target condition. Indeed, a paired groups t-test was highly significant (*P* = 0.0016; means: 14.41 for the left-eye-to-target condition and 3.45 for the right-eye-to-target condition).

**Figure 6.**
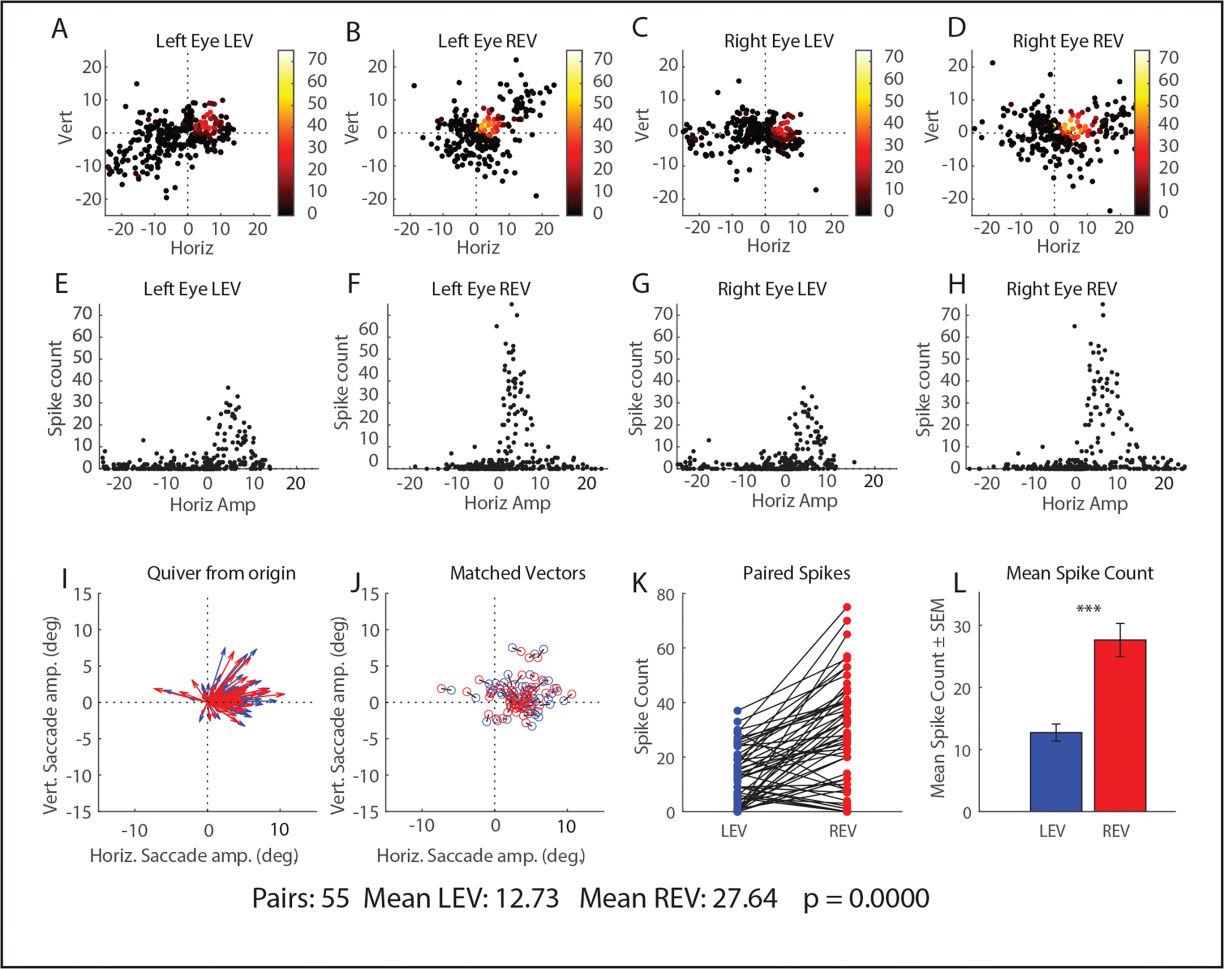
Data for an example neuron, recorded from monkey XT2. Same format and same conventions as Figure 5: Top row (panels **A**–**D**): horizontal and vertical amplitudes of all trials for this neuron. Each dot represents a single saccade, color-coded according to the number of spikes in the burst. Note that there are numerous trials at, and near the movement field peak in both conditions. *Middle row* (panels **E**–**H**): Scatterplots showing the number of spikes as a function of horizontal amplitude for each eye and for both conditions. Note that, even with no analyses, it is straightforwardly obvious that the strongest bursts contain more spikes in the right-eye-to-target condition, compared to the strongest bursts in the left-eye-to-target condition. *Bottom row*: panels **I**–**J** show the vectors of the matched pairs that were included in this analysis. Panel **K** shows the number of spikes for each condition for the matched-vector pairs. The *black lines* connect the values for matched vector pairs. Panel **L**: bar graphs compare the mean number of spikes for matched-vector pairs for the two conditions. Once again, it is clear that the movement field peak was well represented in both conditions (*top row*) and that the strongest bursts included more spikes in the right-eye-to-target condition than the strongest bursts in the left-eye-to-target condition. Again, it is clear from the matched-vector analysis (*bottom row*) that the neuron bursts more strongly for the right-eye-to-target condition than for vector-matched saccades in the left-eye-to-target condition.

Similarly, Figure 6 shows these results for another example neuron, recorded from monkey XT2 (same format as Fig. 5). Note that, for a monkey with large angle exotropia, it is difficult to collect rightward saccades with amplitudes >∼12° for which the left eye was on target both before and after the saccade, because large rightward target steps with the left eye viewing tend to elicit fixation-switching saccades (which were excluded).^59^ Nonetheless, one can see from this figure that the range of data collection included the peak in both conditions and that the number of spikes was clearly greater in the right-eye-to-target condition (Fig. 6H vs. 6E). Figures 6K and 6L confirm this effect for pairs of vector-matched saccades. In the paired groups *t*-test, the matched-vector analysis was highly significant (*P* < 0.00001: means: 12.73 for the left-eye-to-target condition and 27.64 for the right-eye-to-target condition).

**Figure 7.**
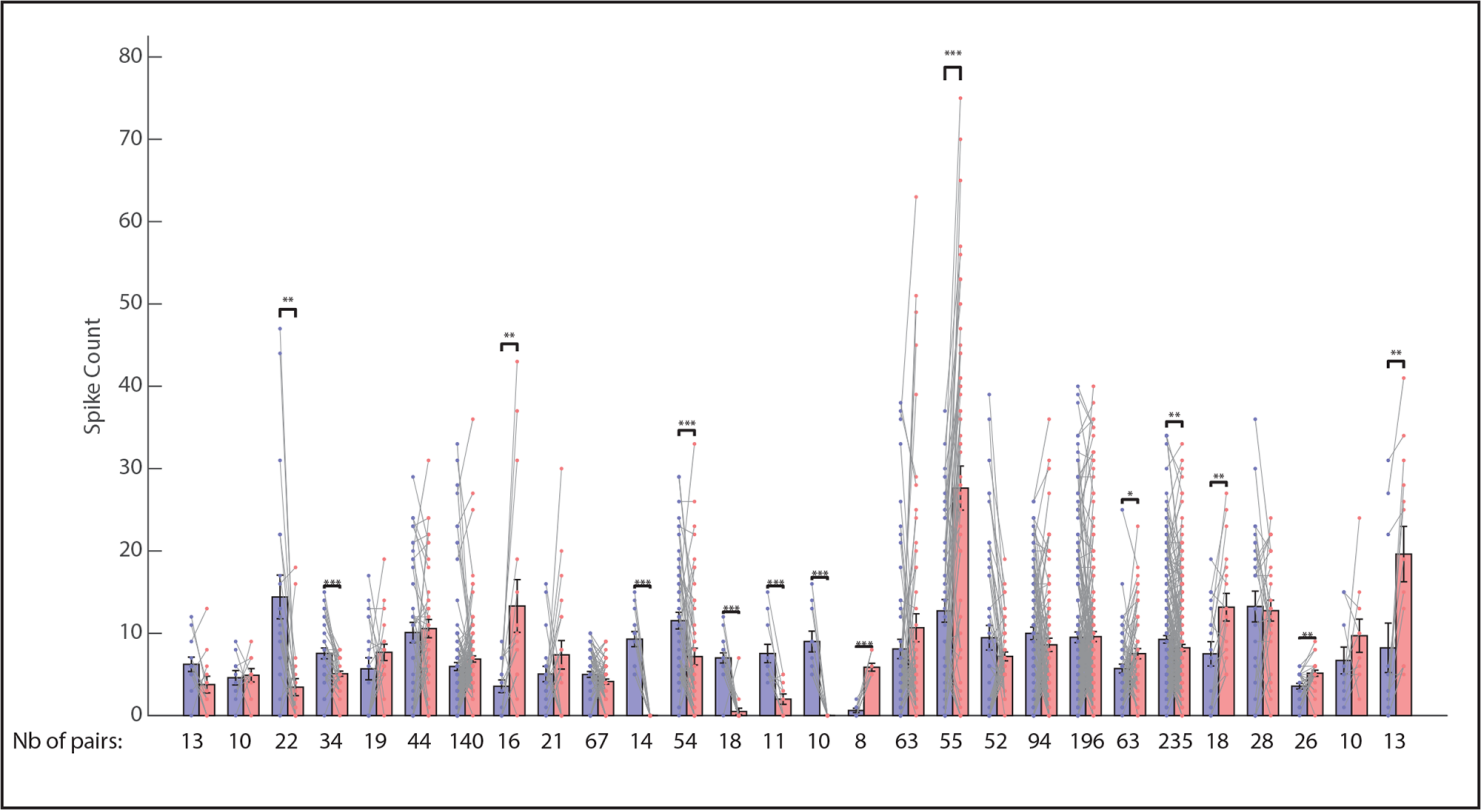
Results of the matched vector analysis for all neurons for which a sufficient number of trials were available (*n* = 29). *P < 0.05 (paired groups *t*-test); **P < 0.01; ***P < 0.001. Overall, a significant difference was found for 15/29 neurons (52%). *Blue bars* represent saccades that brought the left eye to the target; red bars represent saccades that brought the right eye to the target.

Across our sample of neurons 15/29 (52%) showed a significant difference between the two viewing eye conditions in the paired-groups t-tests for the matched vector analysis (Fig. 7). This is a lower percentage of neurons that showed a significant effect, compared to the Gaussian fits, which should not be surprising, given that the matched vector analysis inevitably was based on a much smaller number of trials. Nonetheless, for 13 of the 15 that did show a significant effect, a significant difference was also found for both the “top 10%” analysis and for the Gaussian fits (always in the same direction). For the remaining 2/15, the effect was small for the matched vector analysis, and the Gaussian and top 10% analyses failed to reach significance.

A potential limitation of the matched vectors analysis is that it relies on the assumption that the movement peaks are in the same locations for right-eye-to-target and left-eye-to-target saccades. As noted in the introduction there are reasons to think that this assumption may not be valid. For saccade-related SC neurons recorded during disjunctive saccades in normal monkeys, movement field peaks are often in different locations depending on the vergence demand for the trial.^54^ From a theoretical point of view, any downstream abnormalities that affect the conjugacy of saccades (including eye muscle abnormalities,^1^^,^^2^ misplaced muscle pullies,^60^^,^^61^ or brainstem abnormalities^42^^,^^43^^,^^45^^,^^62^ that affect the saccadic commands for the two eyes differently) has the potential to change the relationship between the saccade requested by SC and the one that is actually observed.

With this possibility in mind, it was investigated whether movement field peak locations were in the same place for right-eye-to-target and left-eye-to-target saccades. The Gaussian functions described above also yield estimates for the horizontal and vertical locations of the peaks for each eye and for each viewing eye condition. These estimates were used to measure the differences in the locations of movement field peaks between left-eye-to-target saccades and right-eye-to-target saccades. In particular, we were interested in the vectorial amplitude of the movement field shift for each neuron. Because the distribution of shift amplitudes is highly skewed, we report the median amplitude of the shift for each neuron: (2.23° for the left eye and 1.6° for the right eye). Although this seems small, one should bear in mind that some of our neurons were recorded from rostral locations, where movement fields tend to be very small. For these neurons, a shift of 1° or 2° may actually be associated with a measurable difference in the burst. Some of our neurons (*n* = 6 for both eyes) showed movement field shifts in excess of 5°. For these reasons, we consider that the Gaussian fits and the “top 10%” analyses are likely to be more valid for these data. Nonetheless, it is worth pointing out that all three methods of comparing the strength of the bursts gave similar answers.

**Figure 8.**
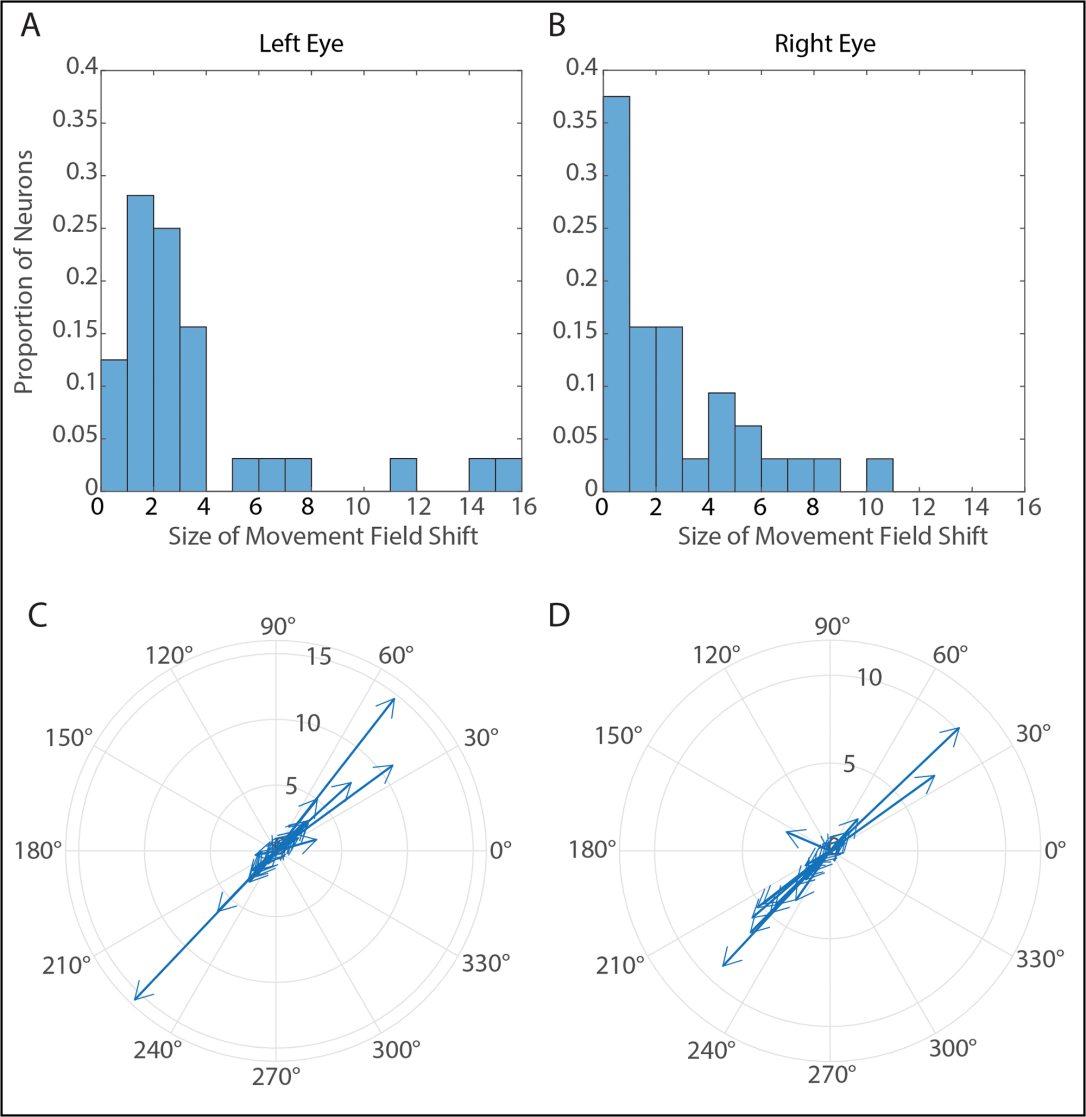
Movement field shifts for saccade-related SC neurons for left-eye-to-target saccades compared to right-eye-to-target saccades. **(A)** Histogram showing the distribution of movement field shifts for the left eye. **(B)** Histogram showing distribution of movement field shifts for the right eye. **(C, D)** Compass plots showing the directions and amplitudes of movement field shifts for the left eye **(C)** and right eye **(D)**.

For an example of one of the movement field shifts, the reader is encouraged to closely examine the Gaussian plots shown in Figures 2A and 2B. In Figure 2A, if one follows the line from the 5° tick mark on the x-axis (horizontal amplitude), one can see that it goes right through the very peak of the movement field. In Figure 2B, one can see that the line from the 5° tick mark passes near the very edge of the movement field. Indeed, in panel B, the movement field peak is at 8° of horizontal amplitude. Figures 8A, 8B show histograms that depict the vectorial amplitude of the movement field shift for the left and right eyes, respectively.

One might worry that what appear to be shifts in the locations of movement field peaks might actually be an artifact of poor Gaussian fits. Indeed, we have previously reported that a subset of neurons in PPRF,^44^ interstitial nucleus of Cajal,^43^ and nucleus prepositus hypoglossi^45^ show abnormally coarse tuning (as measured by *R*^2^ values of curve fits) in strabismus. If this is the explanation for the apparent movement field shifts in the present data set, one would expect to see a tendency for the largest shifts to be associated with the lowest R^2^ values for these fits. However, when we excluded three neurons with very poor fits (*R*^2^ < 0.3) we found no relationship between the measured size of the movement field shift and the *R*^2^ value for the Gaussian fits (*R*^2^ < 0.1 for both the left and right eyes). Figures 8C and 8D show the amplitudes and directions of the movement field shifts for the left and right eyes, respectively. Interestingly, nearly all of the movement field shifts are either up-right or down-left. Although the reasons for this are unclear, it does strongly suggest that the shifts are a real phenomenon and not simply noise related to inadequate goodness-of-fit for the Gaussian curve fits.

We also wondered whether neurons recorded from more caudal locations would show larger movement field shifts than those recorded from more rostral locations. Again, we found no significant correlation between the amplitude of saccades to the movement field peak and the vectorial amplitude of the shift (*R*^2^ < 0.1 when “amplitude of peak” was measured for both the left and right eyes, for both left-eye-to-target and right-eye-to-target saccades).

In previous studies of SC that used monkeys with normal eye alignment, several different types of neurons have been described, including visual cells, visuomotor cells, visually-triggered motor cells, buildup or prelude neurons, and saccade-related burst neurons.^4^^,^^30^^,^^31^^,^^36^^,^^38^^,^^63^^–^^67^ This raises the question of whether the apparent eye selectivity of saccade-related neurons might be solely the result of a loss of the visual component of the response for visuo-motor cells and visually-triggered motor cells, when the target falls within the receptive field for the non-preferred eye. Unfortunately, most of the recordings used for this study were not originally made with this question in mind, and the target step task is not ideal for distinguishing between the various saccade-related cell types in SC. Nonetheless, because visual responses in SC typically begin ∼60 ms after target appearance^38^ and typical saccade latencies in monkeys are close to 200 ms, one can often identify “likely” saccade-related burst neurons, visuomotor cells, and prelude neurons using this task. For example, during performance of the target step task, some of the neurons (designated as “likely VM” cells) showed two quite distinct bursts, one beginning ∼60 ms after the target step, and one that was time-locked to saccade onset. Others (“likely prelude” cells) began to discharge ∼60 ms after the target step and then showed a gradual “buildup” of activity leading up to the saccade. Still others (“likely SRBNs”) showed only a vigorous burst of spikes that was time-locked to saccade onset and did not show evidence of a visual response (i.e. there was no activity that was time-locked to the appearance of the visual target; for visuomotor SC neurons the latency to visual responses is typically ∼60 ms). Of these, 7/8 “likely VM” cells showed significantly stronger bursts for saccades that brought a particular eye to the target (based on the Gaussian fits). This was the case for 7/14 “likely prelude” cells and 9/10 “likely SRBNs.” Thus, although this result should be regarded as somewhat tentative and needs to confirmed by future studies, it does not seem to be the case that the effects described in the present study are solely the result of an eye-specific visual response.

## Discussion

These data show that, for monkeys with strabismus, a subset of saccade-related neurons in SC show stronger bursts (i.e., more spikes) for saccades that bring a particular eye to the target, compared to saccades that bring the other eye to the target. One can clearly see that this is so from the figures, and, importantly, *we are making no other claim in this article*.

That said, there are several possible interpretations of this result. Although the present data are insufficient to show which of these possibilities (if any) is correct, it is hoped that the discussion below may help to guide future studies.

### Possible Interpretation 1 (Unmasking of Monocularity in the Saccadic System)

One possible interpretation is that this is the same phenomenon as the eye-specificity that has been reported in normal animals for brainstem structures, including pontine paramedian reticular formation, central mesencephalic reticular formation, and nucleus prepositus hypoglossi.^25^^,^^27^^,^^68^^–^^70^ If this is the case then the inability to bring both eyes to the same target in subjects with strabismus may simply “unmask” an eye-selectivity that is a normal feature of the saccadic system.

In a 2003 study, Walton and Mays^54^ recorded from saccade-related neurons in SC of normal monkeys although they made saccades between targets that differed in direction (conjugate saccades) as well as between targets that differed in both direction and distance (disjunctive saccades). The main result was that most neurons in their sample showed weaker bursts (i.e., fewer spikes) if the saccade was accompanied by a change in vergence angle. Because humans and monkeys with infantile strabismus syndrome are generally unable to perform disparity vergence movements,^71^^,^^72^ it is somewhat difficult to know whether the present results represent a version of the same phenomenon. Several studies have shown that, in monkeys with infantile strabismus, near and far response cells in the supraoculomotor area modulate their firing rates depending on which eye is used to view the target.^20^^,^^21^^,^^73^ It has also been reported that SC contains neurons that play a role in vergence.^74^ Microstimulation of this area in monkeys with strabismus leads to changes in the angle of strabismus.^75^ These results hint at a possible connection to the present results, but the currently available data are not sufficient to resolve this issue.

Importantly, it must be remembered that, when normal primates make gaze shifts between targets that differ in both direction and distance, both eyes are brought to the same target, even though the eye movements themselves are disjunctive. Consider the neuron shown in Figures 2A and 2B: in theory this neuron could be 100% monocular, driving only the right eye, and still burst vigorously when the left eye goes to the target (because the right eye still moves—and, on some left-eye-to-target saccades, the right eye would still make a saccade to the center of the neuron's movement field). Thus the idea that the existing literature demonstrates the existence of monocular saccade-related cells in normal monkeys^22^^–^^26^^,^^68^^,^^69^ does not inevitably mean that, in monkeys with strabismus, the level of activity in saccade-related SC neurons must vary depending on which eye is brought to the target. For this reason, the present results are still novel, even taking into account the above-cited studies that demonstrate monocular saccade-related neurons in normal monkeys.

Previous studies using non-human primates with strabismus that was induced in infancy have also shown a substantial reduction in the number of binocularly responsive visual neurons in V1,^15^^–^^17^ MT,^18^ and MST.^19^ As noted above, the same neurons that encode symmetrical slow vergence in normal animals have been shown in strabismus to show different levels of tonic activity depending on which eye the monkey is using to view the target.^20^^,^^21^^,^^73^ These studies provide clear evidence for neurophysiological abnormalities in a non-human primate model of infantile strabismus. For these reasons, one should be cautious about assuming that the effect we report in this study is necessarily the same phenomenon as what has been reported in the brainstem in normal animals performing a disjunctive saccade task. To be clear: it might be, but the present study was not designed to address this specific question. This issue will require further research to resolve.

Given the above-cited body of literature showing abnormal loss of binocularity in visual cortex in strabismus, and the well-known facultative suppression of visual information from the non-viewing eye, it should not be surprising for visuomotor neurons in SC to show differing levels of activity, depending on which eye is brought to a visual target. Indeed, this may indicate one mechanism by which abnormalities in visual areas might disrupt the development of normal tuning in oculomotor areas.^1^ Previous reviews of the strabismus neurophysiology literature have discussed, in detail, the possibility that the loss of binocularity in visual cortex leads to a cascade of abnormalities that disrupts development of oculomotor systems. The interested reader is referred to these articles for a much more extensive discussion than is feasible here.^1^^,^^2^

### Possible Interpretation 2 (Extension of the Normal Role of SC in Target Selection)

It is interesting to consider the possibility that the brain might make use of viewing-eye-selective neurons in SC to minimize the adverse consequences of a chronic misalignment of the eyes. A chronic misalignment of the eyes forces the brain to solve a problem that does not typically exist in normal primates, namely the decision of which eye to bring to a visual target. Of course, spatial patterns of visual suppression,^48^ such as visual deficits for targets that activate the temporal hemiretina in exotropia, play a role in this but even subjects with large-angle exotropia typically are able to direct either eye to visual targets in some portions of the visual field (typically target locations that are near straight ahead). One recent study recorded visual and saccade-related activity for SC neurons when a monkey had the opportunity to bring either eye to a visual target.^58^ They reported that visual and buildup activity was greater when the neuron's “preferred vector” was executed, compared to fixation-switching trials in which the other eye's saccade (a non-preferred vector) was executed instead. To the best of our knowledge, these authors did not explicitly compare the overall strength of the burst for right-eye-to-target versus left-eye-to-target saccades but their results do seem consistent with the hypothesis that, in subjects with strabismus, SC activity plays a role in determining *which* eye is brought to a visual target. The idea here is that this would be merely an extension of the role of saccade-related SC neurons in target selection, as has been well documented in normal primates.^31^^,^^32^^,^^39^^,^^67^^,^^76^^–^^78^

### Possible Interpretation 3 (Context-Specific Saccade Adaptation)

As noted above, the amplitude and directional disconjugacy of saccades in strabismus creates a computational problem that the brain must somehow solve. Figure 1 schematically illustrates the issue. According to one widely held view, saccade-related neurons in SC carry information related to desired displacement in normal primates.^40^^,^^79^^,^^80^ So, in this example, the desired displacement is (10,0) but, because the saccades are disconjugate, quite different saccadic commands are needed, depending on which eye is to be brought to the target. It seems likely that this is accomplished through context-specific saccadic adaptation. Previous studies have shown that the brain is thus able to generate, and store, different adaptive states for various contexts such as orbital eye position,^81^^–^^83^ target distance,^84^ and characteristics of the visual target.^85^ If structures such as SC carry information about which eye should be brought to a visual target this could, in principle, provide the information necessary for the brain to establish, and maintain, different adaptive corrections for the two eyes to ensure that the intended eye lands near the target. However, because saccadic adaptation is conjugate in strabismus,^86^ this would not correct the disconjugacy.

Ultimately, we cannot exclude the possibility that none of these interpretations is correct. On the other hand, they are not mutually exclusive so it is also possible that more than one of the above possibilities is correct. Importantly, the present data are not adequate to resolve this issue, so we will confine ourselves to the more limited conclusion that, for many SC neurons, the number of spikes in the burst differs, depending on which eye is brought to the target.

The effect reported in the present study was a somewhat serendipitous discovery so, unfortunately, the behavioral task was not ideal to address the question of whether the various classes of saccade-related neuron in this structure differ in terms of their selectivity for saccades that bring a particular eye to the target. A delayed saccade task and a larger sample size would make it easier to address the more focused question of whether visuomotor cells, saccade-related burst neurons, and neurons with prelude activity show similar difference in the number of spikes depending on which eye is brought to the target. However, a previous study suggested a monocular preference of neurons in the rostral part of the Superior Colliculus despite using a target blink paradigm.^87^

As noted in the methods section, we used prism rearing to induce strabismus in one animal and medial rectus tenotomy in two others. These methods have been used extensively in the literature to induce strabismus in infant monkeys; the result is a permanent strabismus with characteristics that closely match those in human subjects. While we cannot exclude the possibility that eye muscle abnormalities resulting from these surgeries contribute directly to disconjugacy in these animals, the existing literature shows that saccade disconjugacy in these animals is at least partially due to abnormal development of brainstem structures involved in eye movements (for two recent reviews that discuss these issues in detail, please see references 1 and 2).

In summary we report for the first time that, in monkeys with strabismus induced in infancy, a subset of saccade-related neurons in SC burst more strongly for saccades that bring a particular eye to the target, compared to saccades that bring the fellow eye to the target.
